# Insights into the Molecular Mechanism of Endothelial Glycocalyx Dysfunction during Heart Surgery

**DOI:** 10.3390/cimb46050236

**Published:** 2024-04-23

**Authors:** Antea Kršek, Lara Batičić, Božena Ćurko-Cofek, Tanja Batinac, Gordana Laškarin, Silvija Miletić-Gršković, Vlatka Sotošek

**Affiliations:** 1 Faculty of Rijeka, University of Medicine, Braće Branchetta 20, 51000 Rijeka, Croatia; antea.krsek@uniri.hr; 2 Department of Medical Chemistry, Biochemistry and Clinical Chemistry, Faculty of Medicine, University of Rijeka, Braće Branchetta 20, 51000 Rijeka, Croatia; 3 Department of Physiology, Immunology and Pathophysiology, Faculty of Medicine, University of Rijeka, Braće Branchetta 20, 51000 Rijeka, Croatia; bozena.curko.cofek@uniri.hr (B.Ć.-C.); gordana.laskarin@uniri.hr (G.L.); 4 Department of Clinical Medical Sciences I, Faculty of Health Studies, University of Rijeka, Viktora Cara Emina 2, 51000 Rijeka, Croatia; tanjabatinac@net.hr (T.B.); vlatkast@uniri.hr (V.S.); 5 Hospital for Medical Rehabilitation of Hearth and Lung Diseases and Rheumatism “Thalassotherapia-Opatija”, M. Tita 188, 51410 Opatija, Croatia; silvija.miletic@gmail.com; 6 Department of Anesthesiology, Reanimatology, Emergency and Intensive Care Medicine, University of Rijeka, Braće Branchetta 20, 51000 Rijeka, Croatia

**Keywords:** cardiology, endothelial glycocalyx (EGC), endothelial dysfunction, vascular endothelium, vascular health

## Abstract

The endothelial glycocalyx (EGC) is a layer of proteoglycans (associated with glycosaminoglycans) and glycoproteins, which adsorbs plasma proteins on the luminal surface of endothelial cells. Its main function is to participate in separating the circulating blood from the inner layers of the vessels and the surrounding tissues. Physiologically, the EGC stimulates mechanotransduction, the endothelial charge, thrombocyte adhesion, leukocyte tissue recruitment, and molecule extravasation. Hence, severe impairment of the EGC has been implicated in various pathological conditions, including sepsis, diabetes, chronic kidney disease, inflammatory disorders, hypernatremia, hypervolemia, atherosclerosis, and ischemia/reperfusion injury. Moreover, alterations in EGC have been associated with altered responses to therapeutic interventions in conditions such as cardiovascular diseases. Investigation into the function of the glycocalyx has expanded knowledge about vascular disorders and indicated the need to consider new approaches in the treatment of severe endothelial dysfunction. This review aims to present the current understanding of the molecular mechanisms underlying cardiovascular diseases and to elucidate the impact of heart surgery on EGC dysfunction.

## 1. Introduction

The vascular endothelium is the largest organ in the body, which spreads along the luminal side of blood vessels and lymphatic vessels; for decades, it was considered a mechanical barrier separating the circulating blood from the inner layers of the vessels and the surrounding tissues [1,2,3]. The endothelial layer consists of endothelial cells, which produce components of the thin EGC layer extending from their luminal surface [4,5]. The extracellular parts of transmembrane glycoproteins and proteoglycans are anchored in the cell membrane of endothelial cells and form the fundamental structure (matrix) of the EGC, whereas the transmembrane parts of their molecules attach the EGC to endothelial cells [5,6].

**Figure 1:**
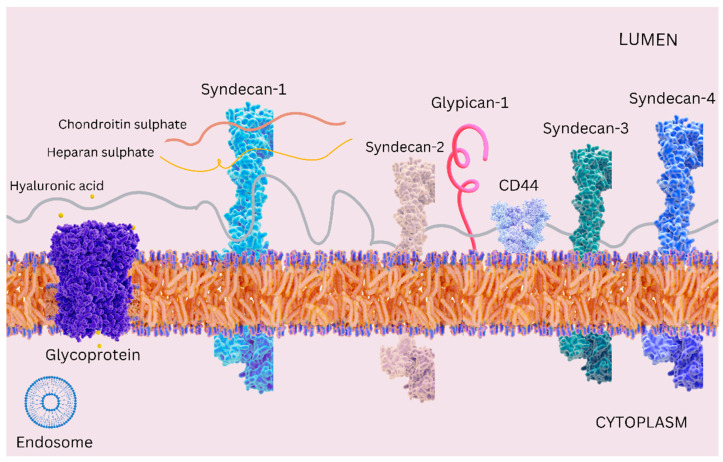
A simplified schematic representation of the glycocalyx, with a focus on cell-surface core proteins, including CD44, glypican-1, syndecan-1, syndecan-2, syndecan-3, and syndecan-4, along with their associated glycosaminoglycans, such as heparan sulfate, chondroitin sulfate, and hyaluronic acid.

Glycoproteins contain short carbohydrate side chains capped with sialic acid in their extracellular parts, which give them a negative charge important for the control of endothelial permeability, as filtration of negatively charged plasma proteins in the extracellular space is diminished [7]. Due to its negative charge, the EGC acts as a barrier against pathogens, deterring bacteria and viruses, as well as participates in controlling platelet interactions, leukocyte adhesion, endothelial permeability, coagulation, and the regulation of vascular tone [5,8,9]. Similarly, proteoglycans (syndecan-1 and glypican-1) expressed on endothelial cells covalently bind negatively charged glycosaminoglycans (chondroitin sulfate, heparan sulfate, and dermatan sulfate) with the extracellular parts of their molecules in different proportions and compositions [5], which contributes to the negative charge of the EGC. Chondroitin sulfate binds mainly to syndecan-1, and heparan sulfate accounts for 50–90% of all glycosaminoglycans bound to syndecan-1 and glypican-1; therefore, these essentially define the properties of the EGC matrix [10] (Figure 1).

The matrix of the EGC includes intracellular adhesion molecule (ICAM)-1, vascular cell adhesion molecule (VCAM)-1 and platelet endothelial cell adhesion molecule (PECAM)-1, also known as CD31, which are responsible for thrombocyte and leukocyte aggregation and leukodiapedesis and which protect endothelial cells from direct exposure to blood cellular elements, thus contributing to the control of inflammation and hemostasis. Heparan sulfate exerts anticoagulant activity [11]. It also binds to proinflammatory mediators (cytokines, chemokines, and growth factors) and morphogens in the circulation and protects them against proteolysis [12]. Heparan-sulfate-containing proteoglycans act as coreceptors for various tyrosine-kinase-based receptors [13], implying their influence on signal transduction in endothelial cells mediated by proinflammatory cytokines and growth factors, lowering their activation threshold or changing the duration of the signal transduction. On the other hand, heparan-sulfate-containing proteoglycans act as endocytic receptors for the clearance of bound lipoproteins or morphogens; in this way, the EGC regulates tissue development/remodeling [12].

**Figure 2:**
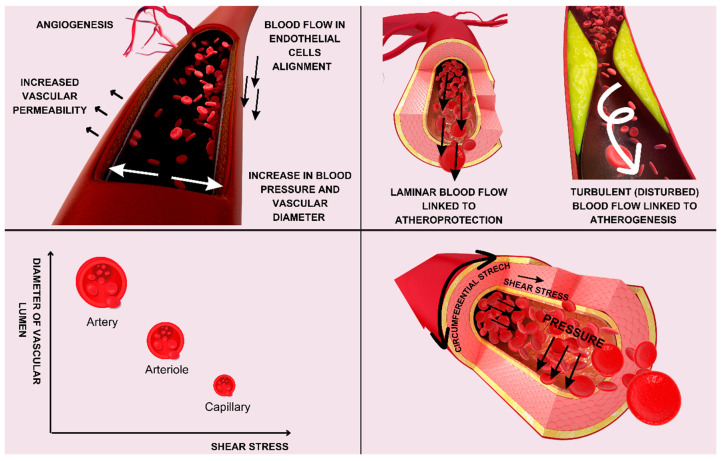
Hemodynamic forces and endothelial mechanosensing acting on endothelial cells.

The EGC plays a crucial role in modulating endothelial functions through various mechanisms, including mechanotransduction, barrier function regulation, leukocyte adhesion modulation, and signaling pathway regulation [1]. The glycocalyx acts as a mechanosensor, converting mechanical forces exerted by the blood flow into biochemical signals, especially in downstream signaling pathways. Recent research suggests that the EGC has an important mechanosensing function [1,14,15,16,17]. Components of the EGC participate in mechanotransduction, as they participate in the translation of shear stress into electrical, functional, and genetic changes inside endothelial cells by connecting the cytoskeleton and cell membrane. Endothelial cells are under the strong influence of hemodynamic forces and other important factors (presented in Figure 2) and exhibit accentuated mechanosensing.

The EGC contributes to the shear-induced signaling pathway through endothelial-cell junctional proteins, such as PECAM-1, a cell-adhesion molecule and endothelial-cell mechanosensor that acts in a complex of different junctional proteins composed of PECAM-1, vascular endothelial cadherin (VE-cadherin), and vascular endothelial growth factor receptor 2 (VEGFR2), which play a central role in endothelial mechanosensing and alignment [16,17,18]. PECAM-1 is involved in mechanotransduction through its interactions with other proteins and its localization in specialized structures within endothelial cells, such as at intercellular junctions and focal adhesions. PECAM-1 plays a multifaceted role in endothelial mechanotransduction by detecting mechanical signals, mediating intercellular communication, regulating focal-adhesion dynamics, and modulating intracellular signaling pathways as well as inflammatory modulation. Mechanical forces generated during leukocyte adhesion and migration can influence PECAM-1-mediated signaling, regulating the inflammatory phenotype of endothelial cells [1,14,15,16,17,18].

Blood flow and shear stress increase the force on PECAM-1, initiating a signaling cascade leading to eNOS and NO production [17]. PECAM-1 has a central role in the response during abrupt changes of flow and in mediating shear-stress-induced vasodilatation, facilitating eNOS activation; it does this by enhancing its activity and through physical interaction at the junction between endothelial cells [16,17,18]. Studies have suggested that the proteoglycan core protein glypican-1 transmits the fluid shear force sensed by glycosaminoglycan (GAG) side chains mainly via heparan sulfate (HS), but not chondroitin sulfate and syndecan-1, to the cell surface, leading to the phosphorylation of eNOS to NO [17,18].

Applying stress directly to glypican-1, rather than PECAM-1, induced nitric oxide (NO) production by human umbilical-vein endothelial cells (HUVECs) and rat fat-pad endothelial cells in vitro. In this model, the generation of NO was abolished by using small interfering RNA (siRNA) to knock down PECAM-1 expression, but also by using glypican-1 knockout mice with normal levels of PECAM-1 expression [18]. These findings highlight glypican-1 as the primary shear sensor upstream and underscore PECAM-1 as the downstream mediator in shear-induced NO generation [18,19,20,21]. Furthermore, heparan sulfate is crucial for initiating early-phase endothelial mechanotransduction by association with PECAM-1 [16], as PECAM-1 activation generates shear-induced NO through the phosphorylation of endothelial nitric oxide synthase (eNOS) in endothelial cells [21,22]. Similarly, hyaluronan, but not chondroitin sulfate, blocks shear-induced NO production [17,18,21].

It has been shown that in the early-phase mechanotransduction in response to a step change in shear stress, PECAM-1 and G-protein Gαq/11 form a mechanosensitive complex that contains endogenous heparan sulfate proteoglycans with an HS chain that is central for complex assembly and flow response regulation [18,19]. The PECAM-1/Gαq/11 complex bound by HS has been shown to be disrupted in studies using heparinase to cleave HS, altering the early-phase induction of NO production [17,18,21]. In addition, it has been shown that heparinase treatment blocked both the early- and late-phase response of NO production to a step change in shear stress [21,22]. PECAM-1 has a role in shear-induced PGI_2_ production and upregulation of the enzyme cyclooxygenase 2 (COX-2), the precursor to PGI_2_, which has been confirmed by different studies [22,23].

**Figure 3:**
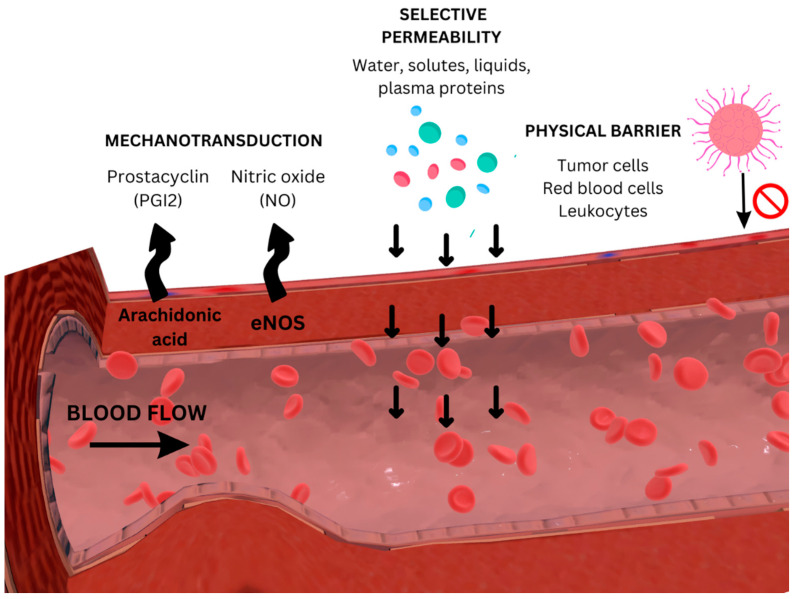
Different roles of the endothelial surface glycocalyx in vascular functions: acting as a mechanosensor, a molecular sieve, and a barrier between circulating cells (e.g., leukocytes, tumor cells) and endothelial cells.

The integrity of the EGC is preserved by physiological blood flow parameters that control the expression of its constituent parts (Figure 3). Under the influence of glycosaminoglycans, water molecules and various plasma proteins are incorporated into the matrix, playing a critical role in maintaining the integrity and hydration of tissues [6,10,23,24,25].

The interaction of plasma proteins (enzymes and cofactors, xanthine-oxidoreductase, superoxide dismutase, and thrombomodulin) with EGC is crucial in maintaining endothelial structure and function [24]. Proteins such as factor H, the C1 inhibitor, and antithrombin III, which bind to heparan sulfate domains within the glycocalyx, participate in vasodilatation and regulate coagulation, preserving vascular hemostasis and health [14].There is a dynamic balance between the layer of soluble EGC components and the circulating blood, which constantly affects the composition and thickness of the glycocalyx, and at the capillary level, they even slow down the blood flow due to their volume [26]. Therefore, the composition, structure, and dimensions of the EGC are quite variable [27]. The thickness of a healthy EGC varies depending on the location within the vascular tree. It represents the foundation required for a robust and effective physiological function of healthy vascular endothelium [26]. Decreased thickness is associated with endothelial dysfunction [28].

Endothelial dysfunction represents a potentially fatal process that manifests in different clinical entities such as ischemic cardiovascular diseases, particularly if they are complicated with diabetes and chronic kidney disease, volume loading (hypernatremia or hypervolemia), hyperkinetic circulation (sepsis), and ischemia/reperfusion damage (for example, during cardiac surgery) [29,30,31]. Additionally, damage to the EGC has been linked to altered outcomes of therapy in cardiovascular diseases [3]. This review aims to present the current understanding of the molecular mechanisms underlying endothelial dysfunction during heart surgery.

## 2. Emerging Insights into the Endothelial Glycocalyx in Cardiovascular Diseases

### 2.1. Atherosclerosis

Vascular function control and endothelial health are significantly impacted by the glycocalyx’s dynamic nature, which is defined by ongoing cycles of degradation and regeneration [32]. Coronary arteries of the White Carneau pigeon had reduced vascular glycocalyx in atheroprone regions [33]. Similarly, the EGC thickness was notably lower in the sinus area of the internal carotid artery in disease-prone mice as compared to the non-diseased sections of the common carotid artery [34]. Van den Berg et al. also noticed a thinner EGC in the branches of the internal carotid artery, with a higher intimal accumulation of low-density lipoproteins (LDLs) than in nearby common carotid regions in mice. This suggests that the increased LDL accumulation is caused by compromised EGC barrier properties [35]. Atherosclerosis-prone Apolipoprotein E-deficient (Apoe^−/−^) mice that were fed a diet rich in cholesterol for 10 weeks showed increased LDL buildup and greater apoptosis in the area of the common carotid artery under the thinner EGC [36]. It was proposed that EGC shedding increased endothelial apoptosis, which increased lipid permeability and promoted monocyte adhesion and subendothelial recruitment, resulting in the formation of atherosclerotic plaques [36,37]. Moreover, selective degradation of the heparan sulfate chains induced the release of cytokines, chemokines, and growth factors [38]. This created an opportunity for the cytokine-mediated activation of leukocytes, resulting in an increase in leukocyte adhesive properties. Subsequently, cell rolling and adhesion during diapedesis were rendered possible by ICAM-1, ICAM-2, VCAM-1, and PECAM-1 on endothelial cells [10]. Additionally, decreased synthesis of glycosaminoglycan hyaluronan promoted leukocyte adhesion, which, in turn, triggered inflammation and accelerated atherosclerosis [37]. This is in accordance with the physiological function of hyaluronan, which interacts with the transmembrane glycoprotein CD44 and prevents its functions, such as adhesion, rolling and diapedesis of leukocyte subpopulations, lymphocyte activation, and angiogenesis [39]. Therefore, the breakdown of hyaluronan via the CD44 molecule promotes endothelial dysfunction and atherosclerosis [40].

Recent investigations demonstrated that partial ligation of the carotid artery in atherosclerosis-prone Apoe^−/−^ mice increase oscillatory shear and microRNA 712 production. This particular microRNA inhibits the tissue inhibitor of MMP 3, leading to the development of atherosclerotic plaques [41,42]. Stained samples of carotid arteries from partial ligation procedures showed the complete lack of hyaluronan [43]. Degradation of hyaluronan [44] and heparan sulfate from proteoglycans inhibited shear-induced NO production. Chondroitin sulfate did not have such an effect [45].

The pathogenesis of heart failure of ischemic etiology in patients has been studied in relation to EGC shedding. Patients with ischemic heart disease or heart failure with preserved ejection fraction (HFpEF) had higher serum syndecan-1 [41] that correlated with the degree of inflammation and faster leukocyte recruitment [46]. Syndecan-1 in patients with HFpEF was associated with the development of acute kidney insufficiency and predicted mortality within 3 years [41]. In patients with heart failure with preserved ejection fraction (HFpEF), the plasma concentration of syndecan-1 was not significantly elevated, whereas serum hyaluronan was considerably higher, and it was an independent predictor of a poorer clinical outcome [47].

### 2.2. Hypertension and Aging

Published data of experimental animal models and humans indicates that the loss of EGC in various vascular compartments is associated with clinical diseases. Rats with diabetes and hypertension exhibited a diminished EGC layer in retinal and choroidal capillaries, whereas spontaneously hypertensive rats showed a thinner EGC layer at the blood–brain barrier [36]. Moreover, increased blood salt levels have been connected to decreased EGC thickness. This decreases the EGC’s capacity to sequester salt, which, in turn, contributes to hypertension and salt overload [48,49]. Blood-borne matrix metalloproteinases (MMPs) cause increased EGC cleavage in the red blood cells of hypertensive rats as opposed to normotensive rats [49]. Schierke et al. have conducted more recent studies that show that increased plasma sodium content, a sign of hypertension, stiffens the endothelium cortex and reduces the thickness of the EGC. This reduction in EGC thickness causes the endothelium to produce proinflammatory cytokines, which, in turn, increases monocyte adhesion and reduces the generation of NO [27]. Rats with monocrotaline-induced pulmonary artery hypertension have been shown to exhibit EGC shedding [50]. In untreated hypertension individuals, an impaired EGC has also been reported that is correlated with arterial stiffness and dysfunction in coronary and cardiac function [51]. Moreover, endothelial shedding has been linked to hypertension associated with preeclampsia, which can result in endothelial dysfunction, decreased microvascular perfusion, and vascular damage [52].

Studies investigating the EGC in microvessels of both young and elderly male mice, as well as in humans, have consistently reported a significant decrease in glycocalyx thickness with aging. This finding underscores the age-related alterations in the EGC, highlighting its potential implications for vascular function and physiology across species [50]. In particular, compared with young mice, the EGC thickness in the mesenteric and skeletal muscle microvessels of old mice was decreased by 51–54%, and the EGC thickness was decreased by 33% in the sublingual microcirculation of elderly people. These results highlight the existence of reduced EGC thickness in older adults together with signs of compromised microvascular perfusion [53].

## 3. Understanding the Effects of Heart Surgery on Vascular Health

Cardiac surgery, involving heart and thoracic aortic surgeries, is necessary to treat the increasing incidence of patients with cardiovascular diseases [54]. Every year, about a million cardiac treatments are performed worldwide [55]. In 2019, guidelines outlining the indications for cardiac surgery were developed in collaboration with the European Association for Cardio-Thoracic Surgery (EACTS), the Quality and Outcomes Committee of the European Board of Cardiovascular Perfusion (EBCP), and the European Association of Cardiothoracic Anesthesiology and Intensive Care (EACTAIC). These indications are mostly related to severe valvular regurgitation or stenosis and advanced ischemic heart disease [56].

For valvular heart disease or for valve repair or replacement, open cardiac surgery may be necessary depending on the damaged valve [57]. If invasive cardiological therapy is not effective for a patient with severe ischemic heart disease, cardiac surgery is recommended [58]. Treatment strategies may involve open or less invasive procedures [59,60].

Most heart treatments involve the use of cardiopulmonary bypass (CPB), which temporarily replaces heart and lung functions with an artificial circuit made up of a pump and an oxygenation barrier [54]. A quiescent heart and a bloodless surgical area are made possible by CPB, which also maintains adequate oxygenation and systemic perfusion. Roller and centrifugal pumps are common components of CPB machines, which offer non-pulsatile flow [14]. While pulsatile flow is recommended in adult open-heart surgery by the 2019 EACTS/EACTAIC/EBCP guidelines, the evidence for its superiority over non-pulsatile flow remains unclear, although pulsatile flow is a more natural option [56,58].

Postoperative organ failure following heart surgery is a result of many mechanisms, including endothelial dysfunction and the release of inflammatory mediators, particularly in heart treatments using CPB [61]. An example of inflammatory mediators generated by surgical stress and chronic cardiac inflammation are interleukins (IL-1, IL-6, IL-8, IL-12, and IL-18) [19,62]. Additionally, surgical stress and volume compensation during and after the procedure lead to the release of atrial natriuretic peptide and damage to the EGC [63]. Mitigating the consequent endothelial dysfunction is a key research focus.

The application of anesthetics, fluid overload, and ischemia–reperfusion damage, which all promote glycocalyx shedding and endothelial dysfunction, are also observed in non-cardiac surgery. However, these changes are more apparent after cardiac surgery due to factors such as endothelial insufficiency, which patients with cardiovascular diseases already have, and longer blood contact with artificial circuits during CPB.

## 4. Emerging Insights into the Endothelial Glycocalyx during Heart Surgery

The intricate endothelial function can be compromised and the EGC damaged during heart surgery due to many factors such as mechanical damage by surgical techniques, acute inflammation, ischemia–reperfusion injury, turbulent blood flow, and contact with foreign chemicals and surfaces [64].

Acute inflammation has a central role in EGC breakdown, as it is the first non-specific and comprehensive defense reaction that tends to limit noxious stimuli and harmful substances during heart surgery. The inflammation is mediated by soluble factors and cells [65]. Activated monocyte/macrophages, neutrophils, and mast cells produce heparanases, hyaluronidases, and matrix metalloproteinases (MMPs) after contact with endothelial matrix proteins [65,66]. Heparanase cleaves heparan sulfate and hyaluronidases cleave hyaluronan from proteoglycans, which promote EGC damage, facilitating adhesion of activated lymphocytes to the surface of endothelial cells and, consequently, leukodiapedesis [66].

The shedding of glycosaminoglycans from EGC facilitates the release of chemokines and proinflammatory cytokines embedded in the EGC into the circulation [66,67,68]. In turn, the proinflammatory cytokines further promote glycocalyx shedding [58,69,70]. Cytokines support the stimulation of lymphocytes, activate complement, and produce reactive oxygen species (ROS) and reactive nitrogen species (RNS), which additionally damage endothelial cells [5,65]. Circulating endothelial cells detached from the basal membrane express TLR4, which activate memory T cells [71]. Circulating endothelial cells possess the capability to identify and present local tissue antigens; however, they may not efficiently activate naive T cells for proliferation and differentiation. Despite this limitation, circulating endothelial cells are able to stimulate these T cells to produce cytokines. This highlights a potential role for endothelial cells in immune regulation through cytokine signaling despite their inability to fully activate T cell proliferation and differentiation [58,72,73,74].

Blood flow causes shear stress of the EGC, which extends into the vessel lumen, especially in constrictions or bifurcations, where flow velocities are higher. Under pressure and shear stress, the endothelium glycocalyx goes through a dynamic process of ongoing breakdown and repair [75]. Magoon et al. highlight the role of a healthy glycocalyx in mechanotransduction, the process by which cells convert mechanical stimuli into biochemical signals [14]. Changes in the glycocalyx that disrupt these interactions can exacerbate disease, emphasizing the importance of the glycocalyx in vascular health [19,20,71]. Glycocalyx shape and thickness differ throughout vascular beds and are highly dependent on shear stress [56,57]. Decreased thickness is associated with vascular dysfunction in different pathophysiological settings [28]. An increase in the concentration of EGC components in the circulation is a sign of its damage and endothelial dysfunction [46]. The destruction of endothelial cells and EGC shedding are major processes involving the metalloproteinase family, in particular MMPs [76,77,78,79]. MMPs 3 and 9 have been linked to cardiovascular diseases, and individuals with ischemic heart disease and atherosclerotic plaques have higher MMP 3 and 9 levels [79,80,81]. Furthermore, MMPs have the ability to cleave hyaluronan and syndecan-1 receptors, which exacerbates glycocalyx damage [5,82,83]. Shed glycocalyx components exacerbate the EGC damage by feeding a vicious cycle of ROS and RNS production [84,85].

EGC shedding following heart surgery is also facilitated by ischemia–reperfusion damage, which releases heparan sulfate and syndecan-1 into the circulation [86,87,88]. Even after off-pump CABG surgery, CPB causes an inflammatory response that increases the shedding of heparan sulfate and syndecan-1 [89].

Research on animals suggests that the endothelium glycocalyx takes five to seven days to restore after shedding [90]. However, clinical investigations have shown that this process occurs much faster [58,91].

## 5. Estimation of Endothelial Glycocalyx Damage in Heart Surgical Settings

In clinical and scientific arenas, conventional laboratory methodologies and biochemical analyses are frequently deployed to assess factors linked to the release of soluble glycocalyx post cardiac surgery [79,92]. It is imperative to comprehend the nuances in circulating molecule levels associated with endothelial shedding. Employing commercially available enzyme-linked immunosorbent assays or enzyme immunoassay kits facilitate the identification and quantification of numerous bioactive molecules, metabolites, cytokines, and other pertinent parameters released or produced by the endothelium. Notable among the frequently studied biomarkers of EGC are angiopoietin-1 and 2, ICAM-1, VECAM-1, P and E-selectin, chondroitin sulfate, hyaluronan, heparan sulfate, and syndecan-1 [62,87,88,92,93,94,95,96,97,98,99]. Target peptides or proteins can have their protein expression levels measured using the Western blot method.

An all-encompassing approach has been devised in the effort to find novel factors linked with microvascular dysfunction. This technique uses sidestream dark-field imaging, which includes imaging with the Gycocheck^TM^ program (Microvascular Health Solutions Inc., Salt Lake City, UT, USA), to perform sublingual video microscopy [100]. Microvascular perfusion and endothelial surface-layer characteristics may be analyzed using this technique, in addition to a number of other variables. They include capillary recruitment, dynamic capillary blood volume, absolute and stationary capillary blood volume, erythrocyte concentration and velocity, blood flow within sublingual microvessels, vascular density, the perfused boundary region (which reflects EGC thickness), and other variables. In addition to all the biological parameters that can be measured, sublingual video microscopy can also provide significant spatial resolution, which refers to the level of detail or the smallest detectable aspects that can be observed in the captured images [100,101,102,103,104,105,^106^]. The spatial resolution achievable with sublingual video microscopy can differ depending on the specific equipment and settings used [101,106]. In general, sublingual video microscopes can provide spatial resolution in the order of micrometers (µm), allowing the visualization of individual capillaries and blood flow dynamics within them. However, spatial resolution in sublingual video microscopy is influenced by several factors; for example, the magnification power, where higher magnification enables the visualization of smaller blood vessels and finer details. Likewise, the optical quality significantly affects spatial resolution, as does the pixel density, which allows finer details to be captured within the field of view, resulting in higher spatial resolution [100,101,106]. Moreover, the size of the area being imaged affects the spatial resolution, as do advanced image-processing techniques that can further enhance spatial resolution by reducing noise, improving contrast, and sharpening edges in the captured images [100,106].

Ikonomidis et al. investigated the relationship between the incidence of cardiovascular events and the disruption of EGC integrity [54,102]. Using the perfused boundary region (PBR5-25) as a proxy for EGC thickness in sublingual microvessels, they discovered that over a 6-year follow-up, cardiovascular events (defined as death, major adverse cardiovascular events, myocardial infarction, and stroke) were significantly predicted by increased PBR5-25, indicating a thinner EGC. Despite the study’s valuable insights, questions were raised regarding the estimate’s stability because there were only 57 events compared with the total number of factors that the research took into account. The criticism argues that more event data and tougher criteria are required for strong prediction capability [103]. Furthermore, the discussion emphasizes the necessity of taking into account the interaction between various serum biomarkers and incorporating validated biomarkers for cardiovascular disease when estimating the mortality risk in patients with heart failure, suggesting that a larger dataset is necessary for precise analysis [104,105].

## 6. Multifaceted Approaches for Preserving Endothelial Glycocalyx Integrity in Heart Surgery

### 6.1. Management of Fluid Dynamics and Protein-Based Therapeutic Approaches

Fluid management is essential for maintaining EGC integrity. Fluid infusion is often necessary to optimize hemodynamic parameters and tissue perfusion, but it is essential to recognize that excessive fluid administration can exacerbate glycocalyx shedding and endothelial damage, potentially worsening vascular permeability and organ dysfunction. Fluid infusion is frequently used in cardiosurgical procedures, but it may increase the concentration of biomarkers associated with EGC shedding [106,107]. Research has demonstrated a steady increase in heparan sulfate concentrations with every liter of intravenous fluid given, suggesting the possibility of iatrogenic endothelial injury [107]. Similar to this, after heart surgery, acute hypervolemic hemodilution can cause EGC damage and mechanical stress, which can result in fluid leakage into the interstitial space. Increased morbidity is correlated with liberal perioperative fluid administration, which produces a positive fluid balance. The evidence that preoperative fluid administration may worsen glycocalyx shedding has cast doubt on this once-common technique. In order to prevent iatrogenic endothelium damage during surgery, rational fluid management that satisfies patients’ clinical demands is advised. As opposed to liberal regimens, implementing restrictive fluid regimens can lower surgical complications and the length of hospital stays [58].

It has been suggested that fresh frozen plasma and human albumin can help regenerate EGC damage. Fresh frozen plasma has protective and regenerative properties and is rich in the plasma proteins required for glycocalyx repair [108,109]. Following a hemorrhage, treatment with fresh frozen plasma has been demonstrated to improve syndecan-1 levels and glycocalyx thickness. It is thought that human albumin, which is commonly used to treat low blood volume, strengthens the glycocalyx structure and reduces shedding [58]. Although research on animals has demonstrated the advantages of albumin, results from clinical trials contrasting it with fresh frozen plasma are still equivocal [110]. However, adding human albumin to solutions decreased EGC shedding and enhanced results in heart transplant models. Furthermore, lactated Ringer’s solution was not as effective as plasma replacement in maintaining glycocalyx parameters in hemorrhagic shock [58].

### 6.2. Sustaining Normal Blood-Glucose Levels

Because both acute and chronic hyperglycemia can harm the EGC and cause cardiovascular problems, maintaining normoglycemia is essential [110,111]. Promising agents for maintaining EGC integrity include metformin and empagliflozin [112,113]. An SGLT2 inhibitor called *empagliflozin*^®^ improves glycocalyx production and repairs endothelial cell responsiveness in EGC injury [112]. Metformin is a commonly used antidiabetic medication that decreases inflammatory indicators and increases glycocalyx thickness and density in addition to lowering glucose levels [114,115,116].

### 6.3. Stabilizers of Atherosclerotic Plaques

For glycocalyx regeneration, atherosclerotic plaque stabilizers such as statins and sulodexide have been proposed [117,118]. The heparan-sulfate source sulodexide has been shown to improve microvascular function and EGC thickness in diabetic patients. Statins, in particular rosuvastatin, have demonstrated encouraging effects in reducing permeability while also increasing EGC thickness [58,117].

### 6.4. Anti-Inflammatory Treatment

Treatments that reduce inflammation may be able to slow the breakdown of the EGC [119,120]. Promising agents for preventing inflammation-induced shedding and preserving the integrity of the EGC include etanercept and hydrocortisone [121]. EGC thickness has also shown positive responses to poloxamer-188, a medication used to treat sickle-cell disease. Analogously, more recent anti-inflammatory medications such as imatinib and tocilizumab have the ability to maintain the integrity of the EGC [122,123].

### 6.5. Anticoagulants

The use of anticoagulant medication, which frequently includes additional antithrombin, is essential in the treatment of disseminated intravascular coagulation brought on by sepsis [124]. Its potential benefits have been extensively researched and include a possible reduction in in-hospital mortality rates as well as the ability to mitigate EGC damage, inhibit the shedding of important components like heparan sulfate and syndecan-1, and prevent vascular leakage in experimental models caused by inflammatory processes like TNF-α infusion or ischemia [125,126]. Furthermore, studies on the antithrombin and enoxaparin combination treatment’s therapeutic effects show encouraging results in lowering leukocyte adhesion and transmigration across the blood–brain barrier after traumatic brain injury, suggesting a possible role in glycocalyx barrier function restoration [127]. There is conflicting evidence when it comes to heparin therapy. On the one hand, unfractionated heparin supplementation in conjunction with crystalloids and antibiotics seems to normalize the EGC and may even have a protective or anti-inflammatory effect [128]. On the other hand, there are worries that it may compete with the heparan sulfate component of the EGC, resulting in structural degradation and compromised barrier function [129]. In addition, low-molecular-weight heparin administered intravenously has been shown to exhibit increased enzymatic activity that releases embedded proteins from the EGC [130,131]. This raises concerns about the EGC aiding the drug’s long-term effects, especially in individuals with diabetes. Anticoagulant medicines therefore hold potential for maintaining the integrity of the EGC. Nevertheless, due to the complex interactions that these therapies have with EGC functions, further research is required to determine their overall therapeutic impact [131].

### 6.6. Anesthetics and Anesthesia Methods

Certain anesthetics, particularly intravenous agents like propofol and volatile inhalational agents such as sevoflurane and isoflurane, have been shown to directly affect EGC integrity. Studies have demonstrated that these agents can lead to glycocalyx shedding and endothelial dysfunction, possibly through mechanisms involving oxidative stress, inflammation, and alterations in intracellular signaling pathways [132,133,134]. Some other studies, particularly in ischemia–reperfusion injuries, have provided evidence that volatile anesthetics such as sevoflurane demonstrate protective effects on the EGC. Clinical evidence supporting their advantage over other anesthetic drugs, however, is still equivocal, and further studies are needed to clarify their benefits or potential harmful effects [132,133,134,135].

## 7. Conclusions

The healthy EGC performs many functions, such as the regulation of mechanotransduction, vascular integrity, vascular tone modulation, NO production, and anti-inflammatory and anti-coagulant activities. Endothelial dysfunction with a damaged glycocalyx represents a generalized vasculopathy with a predominance of vasoconstrictive, procoagulant, and inflammatory properties of the endothelium over its vasodilating, anticoagulant, fibrinolytic, and anti-inflammatory potential.

Vascular diseases are leading causes of mortality and morbidity worldwide. Therefore, any factor preserving EGC structure and function should be thoroughly investigated. Some of those factors are mechanical damage, inflammation, and the formation of blood clots. Any condition, including the procedures during the heart surgery, which causes the shear stress and damage to the EGC leads to pathological changes in the vascular wall, resulting in atherosclerosis, chronic venous disease, hypertension, and aging. Consequently, it leads to coronary heart disease, cerebrovascular disease, and peripheral artery disease, which greatly affect the quality of life, represent a socioeconomic burden, and often have a lethal outcome. Therefore, understanding the physiology and pathophysiology of EGC in health, disease, and surgical procedures is crucial to preventing its damage and maintaining vascular homeostasis.

## References

[1] Reitsma S., Slaaf D.W., Vink H., van Zandvoort M.A., Oude Brink M.G. (2007). The endothelial glycocalyx: Composition, functions, and visualization. Pflugers. Arch..

[2] Starling E.H. (1896). On the Absorption of Fluids from the Connective Tissue Spaces. J. Physiol..

[3] Yilmaz O., Afsar B., Ortiz A., Kanbay M. (2019). The role of endothelial glycocalyx in health and disease. Clin. Kidney. J..

[4] Krüger-Genge A., Blocki A., Franke R.P., Jung F. (2019). Vascular Endothelial Cell Biology: An. Update. Int. J. Mol. Sci..

[5] Foote C.A., Soares R.N., Ramirez-Perez F.I., Ghiarone T., Aroor A., Manrique-Acevedo C., Padilla J., Martinez-Lemus L. (2022). Endothelial glycocalyx. Compr. Physiol..

[6] Pillinger N.L., Kam P. (2017). Endothelial glycocalyx: Basic science and clinical implications. Anesth. Intensive Care.

[7] Moore K.H., Murphy H.A., George E.M. (2021). The glycocalyx: A central regulator of vascular function. Am. J. Physiol. Regul. Integr. Comp. Physiol..

[8] Jedlicka J., Becker B.F., Chappell D. (2020). Endothelial glycocalyx. Crit. Care Clin..

[9] Wang G., Tiemeier G.L., van der Berg B.M., Rabelink T.J. (2020). Endothelial glycocalyx Hyaluronan: Rendothelial glycocalyx ulation and Role in Prevention of Diabetic Complications. Am. J. Pathol..

[10] Cosgun Z.C., Fels B., Kusche-Vihrog K. (2020). Nanomechanics of the endothelial glycocalyx: From structure to function. Am. J. Pathol..

[11] Stachtea X.N., Tykesson E., van Kuppevelt T.H., Feinstein R., Malmström A., Reijmers R.M., Maccarana M. (2015). Dermatan Sulphate-Free Mice Display Embryological Defects and Are Neonatal Lethal Despite Normal Lymphoid and Non-Lymphoid Organogenesis. PLoS ONE.

[12] Sarrazin S., Lamanna W.C., Esko J.D. (2011). Heparan sulphate proteoglycans. Cold Spring Harb Perspect Biol..

[13] Lepedda A.J., Nieddu G., Formato M., Baker M.B., Fernández-Pérez J., Moroni L. (2021). Glycosaminoglycans: From Vascular Physiology to Tissue Engineering Applications. Front. Chem..

[14] Magoon R., Shri I., Das D. (2023). Outcomes Following On-Pump Versus Of-Pump CABG: Apprising the “Bypassed”. Braz. J. Cardiovasc. Surg..

[15] Mahmoud M., Mayer M., Cancel L.M., Bartosch A.M., Mathews R., Tarbell J.M. (2021). The glycocalyx core protein Glypican 1 protects vessel wall endothelial cells from stiffness-mediated dysfunction and disease. Cardiovasc. Res..

[16] Weinbaum S., Cancel L.M., Fu B.M., Tarbell J.M. (2021). The Glycocalyx and Its Role in Vascular Physiology and Vascular Related Diseases. Cardiovasc. Eng. Technol..

[17] Pahakis M.Y., Kosky J.R., Dull R.O., Tarbell J.M. (2007). The role of endothelial glycocalyx components in mechanotransduction of fluid shear stress. Biochem. Biophys. Res. Commun..

[18] Zeng Y., Zhang X.F., Fu B.M., Tarbell J.M. (2018). The Role of Endothelial Surface Glycocalyx in Mechanosensing and Transduction. Adv. Exp. Med. Biol..

[19] Tan A., Newey C., Falter F. (2022). Pulsatile Perfusion during Cardiopulmonary Bypass: A Literature Review. J. Extra. Corpor. Technol..

[20] Villalba N., Baby S., Yuan S.Y. (2021). The Endothelial glycocalyx as a Double-Edged Sword in Microvascular Homeostasis and Pathogenesis. Front. Cell Dev. Biol..

[21] Osawa M., Masuda M., Harada N., Lopes R.B., Fujiwara K. (1997). Tyrosine phosphorylation of platelet endothelial cell adhesion molecule-1 (PECAM-1, CD31) in mechanically stimulated vascular endothelial cells. Eur. J. Cell Biol..

[22] Masuda M., Osawa M., Shigematsu H., Harada N., Fujiwara K. (1997). Platelet endothelial cell adhesion molecule-1 is a major SH-PTP2 binding protein in vascular endothelial cells. FEBS Lett..

[23] Bennett H.S. (1963). Morphological aspects of extracellular polysaccharides. J. Histochem. Cytochem..

[24] Brouns S.L.N., Provenzale I., van Geffen J.P., van der Meijden P.E.J., Heemskerk J.W.M. (2020). Localized endothelial-based control of platelet aggrendothelial glycocalyx ation and coagulation under flow: A proof-of-principle vessel-on-a-chip study. J. Thromb. Haemost..

[25] Kincses A., Santa-Maria A.R., Walter F.R., Dér L., Horányi N., Lipka D.V., Valkai S., Deli M.A., Dér A. (2020). A chip device to determine surface charge properties of confluent cell monolayers by measuring streaming potential. Lab. Chip..

[26] Klitzman B., Dulingg B.R. (1979). Microvascular hematocrit and red cell flow in resting and contracting striated muscle. Am. J. Physiol. Heart Circ. Physiol..

[27] Schierke F., Wyrwoll M.J., Wisdorf M., Niedzielski L., Maase M., Ruck T., Meuth S.G., Kusche-Vihrog K. (2017). Nanomechanics of the endothelial glycocalyx contribute to Na+-induced vascular inflammation. Sci. Rep..

[28] Stevens J.R., Zamani A., Osborne J.I.A., Zamani R., Akrami M. (2021). Critical evaluation of stents in coronary angioplasty: A systematic review. Biomed. Eng. Online..

[29] Kei C.Y., Singh K., Dautov R.F., Nguyen T.H., Chirkov Y.Y., Horowitz J.D. (2023). Coronary “Microvascular Dysfunction”: Evolving Understanding of Pathophysiology, Clinical Implications, and Potential Therapeutics. Int. J. Mol. Sci..

[30] Sukudom S., Smart L., Macdonald S. (2024). Association between intravenous fluid administration and endothelial glycocalyx shedding in humans: A systematic review. Intensive Care Med. Exp..

[31] Chappell D., Bruegger D., Potzel J., Jacob M., Brettner F., Vogeser M., Conzen P., Becker B.F., Rehm M. (2014). Hypervolemia increases release of atrial natriuretic peptide and shedding of the endothelial glycocalyx. Crit Care.

[32] Wang S., Qiu Y., Bai B. (2019). The Expression, Rendothelial glycocalyx ulation, and Biomarker Potential of Glypican-1 in Cancer. Front. Oncol..

[33] Lewis J.C., Taylor R.G., Jones N.D., St Clair R.W., Cornhill J.F. (1982). Endothelial surface characteristics in pigeon coronary artery atherosclerosis. I. Cellular alterations during the initial stages of dietary cholesterol challenge. Lab. Investig..

[34] van den Berg B.M., Spaan J.A., Rolf T.M., Vink H. (2006). Atherogenic rendothelial glycocalyx ion and diet diminish glycocalyx dimension and increase intima-to-media ratios at murine carotid artery bifurcation. Am. J. Physiol. Heart. Circ. Physiol..

[35] van den Berg B.M., Spaan J.A., Vink H. (2009). Impaired glycocalyx barrier properties contribute to enhanced intimal low-density lipoprotein accumulation at the carotid artery bifurcation in mice. Pflugers. Arch..

[36] Cancel L.M., Ebong E.E., Mensah S., Hirschberg C., Tarbell J.M. (2016). Endothelial glycocalyx, apoptosis and inflammation in an atherosclerotic mouse model. Atherosclerosis.

[37] Nagy N., Freudenberger T., Melchior-Becker A., Röck K., Ter Braak M., Jastrow H., Kinzig M., Lucke S., Suvorava T., Kojda G. (2010). Inhibition of hyaluronan synthesis accelerates murine atherosclerosis: Novel insights into the role of hyaluronan synthesis. Circulation.

[38] Vlodavsky I., Barash U., Nguyen H.M., Yang S.M., Ilan N. (2021). Biology of the Heparanase-Heparan Sulphate Axis and Its Role in Disease Pathogenesis. Semin. Thromb. Hemost..

[39] Sneath R.J., Mangham D.C. (1998). The normal structure and function of CD44 and its role in neoplasia. Mol. Pathol..

[40] Kobayashi T., Chanmee T., Itano N. (2020). Hyaluronan: Metabolism and Function. Biomolecules.

[41] Kim Y.H., Nijst P., Kiefer K., Tang W.H. (2017). Endothelial glycocalyx as biomarker for cardiovascular diseases: Mechanistic and clinical implications. Curr. Heart Fail. Rep..

[42] Son D.J., Kumar S., Takabe W., Kim C.W., Ni C.W., Alberts-Grill N., Jang I.H., Kim S., Kim W., Won Kang S. (2013). The atypical mechanosensitive microRNA-712 derived from pre-ribosomal RNA induces endothelial inflammation and atherosclerosis. Nat. Commun..

[43] Tarbell J.M., Cancel L.M. (2016). The glycocalyx and its significance in human medicine. J. Intern. Med..

[44] Mochizuki S., Vink H., Hiramatsu O., Kajita T., Shigeto F., Spaan J.A., Kajiya F. (2003). Role of hyaluronic acid glycosaminoglycans in shear-induced endothelium-derived nitric oxide release. Am. J. Physiol. Heart Circ. Physiol..

[45] Pahakis M., Kosky J., Tarbell J. Sialic acids And Heparan Sulphate Proteoglycans Are Mechanosensory Components of the Endothelial Cell Glycocalyx. Proceedings of the 2005 Summer Bioengineering Conference.

[46] Gopal S. (2020). Syndecans in Inflammation at a Glance. Front. Immunol..

[47] Nijst P., Cops J., Martens P., Swennen Q., Dupont M., Tang W.H.W., Mullens W. (2018). Endovascular shedding markers in patients with heart failure with reduced ejection fraction: Results from a single-center exploratory study. Microcirculation.

[48] Kusche-Vihrog K., Oberleithner H. (2012). An emerging concept of vascular salt sensitivity. Biol. Rep..

[49] Oberleithner H. (2014). Vascular endothelium: A vulnerable transit zone for merciless sodium. Nephrol. Dial. Transplant..

[50] Guo J., Yang Z.C., Liu Y. (2019). Attenuating Pulmonary Hypertension by Protecting the Intendothelial glycocalyx rity of Glycocalyx in Rats Model of Pulmonary Artery Hypertension. Inflammation.

[51] Ikonomidis I., Voumvourakis A., Makavos G., Triantafyllidi H., Pavlidis G., Katogiannis K., Benas D., Vlastos D., Trivilou P., Varoudi M. (2018). Association of impaired endothelial glycocalyx with arterial stiffness, coronary microcirculatory dysfunction, and abnormal myocardial deformation in untreated hypertensives. J. Clin. Hypertens..

[52] Weissgerber T.L., Garcia-Valencia O., Milic N.M., Codsi E., Cubro H., Nath M.C., White W.M., Nath K.A., Garovic V.D. (2019). Early Onset Preeclampsia Is Associated with Glycocalyx Dendothelial glycocalyx radation and Reduced Microvascular Perfusion. J. Am. Heart Assoc..

[53] Machin D.R., Bloom S.I., Campbell R.A., Phuong T.T.T., Gates P.E., Lesniewski L.A., Rondina M., Donato A.J. (2018). Advanced age results in a diminished endothelial glycocalyx. Am. J. Physiol. Heart Circ. Physiol..

[54] Verrier E.D. (1999). Cardiac surgery. J. Am. Coll. Surg..

[55] Vervoort D., Meuris B., Meyns B., Verbrugghe P. (2020). Global cardiac surgery: Access to cardiac surgical care around the world. J. Thorac. Cardiovasc. Surg..

[56] Wahba A., Milojevic M., Boer C., De Somer F.M.J.J., Gudbjartsson T., van den Goor J., Jones T.J., Lomivorotov V., Merkle F., Ranucci M. (2020). EACTS/EACTA/EBCP Committee Reviewers. 2019 EACTS/EACTA/EBCP guidelines on cardiopulmonary bypass in adult cardiac surgery. Eur. J. Cardiothorac. Surg..

[57] Sirendothelial Glycocalyxar S., Groenwold R.H., de Heer F., Bots M.L., van der Graaf Y., van Herwerden L.A. (2012). Performance of the original EuroSCORE. Eur. J. Cardiothorac. Surg..

[58] Knežević D., Ćurko-Cofek B., Batinac T., Laškarin G., Rakić M., Šoštarič M., Zdravković M., Šustić A., Sotošek V., Batičić L. (2023). Endothelial Dysfunction in Patients Undergoing Cardiac Surgery: A Narrative Review and Clinical Implications. J. Cardiovasc. Dev. Dis..

[59] Giacoppo D., Alfonso F., Xu B., Claessen B.E.P.M., Adriaenssens T., Jensen C., Pérez-Vizcayno M.J., Kang D.Y., Degenhardt R., Pleva L. (2020). Drug-Coated Balloon Angioplasty Versus Drug-Eluting Stent Implantation in Patients with Coronary Stent Restenosis. J. Am. Coll Cardiol..

[60] Institute of Medicine (US) Committee on Social Security Cardiovascular Disability Criteria (2010). Cardiovascular Disability: Updating the Social Security Listings.

[61] Conrad C., Eltzschig H.K. (2020). Disease Mechanisms of Perioperative Organ Injury. Anesth. Analg..

[62] Pesonen E., Passov A., Andersson S., Suojaranta R., Niemi T., Raivio P., Salmenperä M., Schramko A. (2019). Glycocalyx Dendothelial glycocalyx radation and Inflammation in Cardiac Surgery. J. Cardiothorac. Vasc. Anesth..

[63] Song J.W., Goligorsky M.S. (2018). Perioperative implication of the endothelial glycocalyx. Korean J. Anesthesiol..

[64] Dogné S., Flamion B. (2020). Endothelial glycocalyx Impairment in Disease: Focus on Hyaluronan Shedding. Am. J. Pathol..

[65] Ågren M.S., Auf dem Keller U. (2020). Matrix Metalloproteinases: How Much Can They Do?. Int. J. Mol. Sci..

[66] Goetzl E.J., Banda M.J., Leppert D. (1996). Matrix metalloproteinases in immunity. J. Immunol..

[67] Rabia B., Thanigaimani S., Golledge J. (2024). The Potential Involvement of Glycocalyx Disruption in Abdominal Aortic Aneurysm Pathogenesis. Cardiovasc. Pathol..

[68] Hu Z., Cano I., D‘Amore P.A. (2021). Update on the Role of the Endothelial Glycocalyx in Angiogenesis and Vascular Inflammation. Front. Cell Dev. Biol..

[69] Kawahara R., Granato D.C., Yokoo S., Domingues R.R., Trindade D.M., Paes Leme A.F. (2017). Mass spectrometry-based proteomics revealed Glypican-1 as a novel ADAM17 substrate. J. Proteomics..

[70] Yang J., LeBlanc M.E., Cano I., Saez-Torres K.L., Saint-Geniez M., Ng Y.S., D‘Amore P.A. (2020). ADAM10 and ADAM17 proteases mediate proinflammatory cytokine-induced and constitutive cleavage of endomucin from the endothelial surface. J. Biol. Chem..

[71] Pahwa R., Nallasamy P., Jialal I. (2016). Toll-like receptors 2 and 4 mediate hyperglycemia induced macrovascular aortic endothelial cell inflammation and perturbation of the endothelial glycocalyx. J. Diabetes Complicat..

[72] Peng L.P., Cao Y., Zhao S.L., Huang Y.X., Yang K., Huang W. (2019). Memory T cells delay the progression of atherosclerosis via AMPK signaling pathway. Ann. Transl. Med..

[73] Abassi Z., Armaly Z., Heyman S.N. (2020). Glycocalyx Dendothelial glycocalyx radation in Ischemia-Reperfusion Injury. Am. J. Pathol..

[74] Jameson S.C., Masopust D. (2018). Understanding Subset Diversity in T Cell Memory. Immunity..

[75] Ali M.M., Mahmoud A.M., Le Master E., Levitan I., Phillips S.A. (2019). Role of matrix metalloproteinases and histone deacetylase in oxidative stress-induced dendothelial glycocalyx radation of endothelial glycocalyx. Am. J. Physiol. Heart Circ. Physiol..

[76] Yang X., Meendothelial Glycocalyxan J.E., Jannaway M., Coleman D.C., SYuan S.Y. (2018). A disintendothelial glycocalyx rin and metalloproteinase 15-mediated glycocalyx shedding contributes to vascular leakage during inflammation. Cardiovasc. Res..

[77] Cooper S., McDonald K., Burkat D., Leask R.L. (2017). Stenosis Hemodynamics Disrupt the Endothelial Cell Glycocalyx by MMP Activity Creating a Proinflammatory Environment. Ann. Biomed. Eng..

[78] Ramnath R.D., Butler M.J., Newman G., Desideri S., Russell A., Lay A.C., Neal C.R., Qiu Y., Fawaz S., Onions K.L. (2020). Blocking matrix metalloproteinase-mediated syndecan-4 shedding restores the endothelial glycocalyx and glomerular filtration barrier function in early diabetic kidney disease. Kidney Int..

[79] Milusev A., Rieben R., Sorvillo N. (2022). The Endothelial glycocalyx: A Possible Therapeutic Target in Cardiovascular Disorders. Front. Cardiovasc. Med..

[80] Sun H., Zhang J., Zheng Y., Shang S. (2018). Expressions and clinical significance of factors related to acute coronary syndrome. J. Biol. Rendothelial Glycocalyx Homeost. Agents..

[81] Reine T.M., Lanzalaco F., Kristiansen O., Enget A.R., Satchell S., Jenssen T.G., Kolset S.O. (2019). Matrix metalloproteinase-9 mediated shedding of syndecan-4 in glomerular endothelial cells. Microcirculation.

[82] Sieve I., Münster-Kühnel A.K., Hilfiker-Kleiner D. (2018). Rendothelial glycocalyx ulation and function of endothelial glycocalyx layer in vascular diseases. Vascul. Pharmacol..

[83] Lee H., Ibrahimi L., Azar D.T., Han K.Y. (2023). The Role of Membrane-Type 1 Matrix Metalloproteinase-Substrate Interactions in Pathogenesis. Int. J. Mol. Sci..

[84] Hahn R.G., Patel V., Dull R.O. (2021). Human glycocalyx shedding: Systematic review and critical appraisal. Acta Anaesthesiol. Scand..

[85] Jackson-Weaver O., Friedman J.K., Rodriguez L.A., Hoof M.A., Drury R.H., Packer J.T., Smith A., Guidry C., Duchesne J.C. (2019). Hypoxia/reoxygenation decreases endothelial glycocalyx via reactive oxygen species and calcium signaling in a cellular model for shock. J. Trauma Acute Care Surg..

[86] Koning N.J., Vonk A.B.A., Vink H., Boer C. (2016). Side-by-Side Alterations in Glycocalyx Thickness and Perfused Microvascular Density During Acute Microcirculatory Alterations in Cardiac Surgery. Microcirculation.

[87] Wu Q., Gao W., Zhou J., He G., Ye J., Fang F., Luo J., Wang M., Xu H., Wang W. (2019). Correlation between acute dendothelial glycocalyx radation of the endothelial glycocalyx and microcirculation dysfunction during cardiopulmonary bypass in cardiac surgery. Microvasc. Res..

[88] Bol M.E., Huckriede J.B., van de Pas K.G.H., Delhaas T., Lorusso R., Nicolaes G.A.F., Sels J.E.M., van de Poll M.C.G. (2022). Multimodal measurement of glycocalyx dendothelial glycocalyx radation during coronary artery bypass grafting. Front. Med..

[89] Squiccimarro E., Stasi A., Lorusso R., Paparella D. (2022). Narrative review of the systemic inflammatory reaction to cardiac surgery and cardiopulmonary bypass. Artif. Organs..

[90] Shinohara A., Ushiyama A., Iijima T. (2021). Time-Dependent Dynamics Required for the Degradation and Restoration of the Vascular Endothelial Glycocalyx Layer in Lipopolysaccharide-Treated Septic Mice. Front. Cardiovasc. Med..

[91] Goncharov N.V., Nadeev A.D., Jenkins R.O., Avdonin P.V. (2017). Markers and Biomarkers of Endothelium: When Something Is Rotten in the State. Oxid. Med. Cell Longev..

[92] Passov A., Schramko A., Salminen U.S., Aittomäki J., Andersson S., Pesonen E. (2021). Endothelial glycocalyx during early reperfusion in patients undergoing cardiac surgery. PLoS ONE.

[93] Dekker N.A.M., Veerhoek D., Koning N.J., van Leeuwen A.L.I., Elbers P.W.G., van den Brom C.E., Vonk A.B.A., Boer C. (2019). Postoperative microcirculatory perfusion and endothelial glycocalyx shedding following cardiac surgery with cardiopulmonary bypass. Anaesthesia.

[94] Hadem J., Rossnick R., Hesse B., Herr M., Hansen M., Bergmann A., Kensah G., Maess C., Baraki H., Kümpers P. (2020). Endothelial dysfunction following coronary artery bypass grafting: Influence of patient and procedural factors. Herz.

[95] Girão-Silva T., Fonseca-Alaniz M.H., Ribeiro-Silva J.C., Lee J., Patil N.P., Dallan L.A., Baker A.B., Harmsen M.C., Kriendothelial Glycocalyxer J.E., Miyakawa A.A. (2021). High stretch induces endothelial dysfunction accompanied by oxidative stress and actin remodeling in human saphenous vein endothelial cells. Sci. Rep..

[96] Abou-Arab O., Kamel S., Beyls C., Huette P., Bar S., Lorne E., Galmiche A., Guinot P.G. (2020). Vasoplendothelial glycocalyx ia After Cardiac Surgery Is Associated with Endothelial glycocalyx Alterations. J. Cardiothorac. Vasc. Anesth..

[97] Brettner F., Chappell D., Nebelsiek T., Hauer D., Schelling G., Becker B.F., Rehm M., Weis F. (2019). Preinterventional hydrocortisone sustains the endothelial glycocalyx in cardiac surgery. Clin. Hemorheol. Microcirc..

[98] Hohn A., Baumann A., Pietroschinsky E., Franklin J., Illerhaus A., Buchwald D., Hinkelbein J., Zahn P.K., Annecke T. (2021). Hemoadsorption: Effective in reducing circulating fragments of the endothelial glycocalyx during cardiopulmonary bypass in patients undergoing on-pump cardiac surgery? Minerva. Anestesiologica.

[99] Robich M., Ryzhov S., Kacer D., Palmeri M., Peterson S.M., Quinn R.D., Carter D., Sheppard F., Hayes T., Sawyer D.B. (2020). Prolonged Cardiopulmonary Bypass is Associated with Endothelial glycocalyx Dendothelial glycocalyx radation. J. Surg. Res..

[100] Rovas A., Sackarnd J., Rossaint J., Kampmeier S., Pavenstädt H., Vink H., Kümpers P. (2021). Identification of novel sublingual parameters to analyze and diagnose microvascular dysfunction in sepsis: The NOSTRADAMUS study. Crit. Care.

[101] Rovas A., Seidel L.M., Vink H., Pohlkötter T., Pavenstädt H., Ertmer C., Hessler M., Kümpers P. (2019). Association of sublingual microcirculation parameters and endothelial glycocalyx dimensions in resuscitated sepsis. Crit. Care.

[102] Ikonomidis I., Thymis J., Simitsis P., Koliou G.A., Katsanos S., Triantafyllou C., Kousathana F., Pavlidis G., Kountouri A., Polyzogopoulou E. (2022). Impaired Endothelial glycocalyx Predicts Adverse Outcome in Subjects Without Overt Cardiovascular Disease: A 6-Year Follow-up Study. J. Cardiovasc. Transl. Res..

[103] Kawada T. (2023). Biomarkers of Endothelial glycocalyx Intendothelial glycocalyx rity for Cardiovascular Events in Individuals Without Cardiovascular Disease. J. Cardiovasc. Transl. Res..

[104] Kim Y.H., Kitai T., Morales R., Kiefer K., Chaikijurajai T., Tang W.H.W. (2022). Usefulness of Serum Biomarkers of Endothelial glycocalyx Damage in Prognosis of Decompensated Patients with Heart Failure with Reduced Ejection Fraction. Am. J. Cardiol..

[105] Kitagawa Y., Kawamura I., Suzuki K., Okada H., Ishihara T., Tomita H., Suzuki K., Takada C., Sampei S., Kano S. (2021). Serum syndecan-1 concentration in hospitalized patients with heart failure may predict readmission-free survival. PLoS ONE.

[106] Long R., Vink H. (2016). (Microvascular Health Solutions LLC). Synergistic Glycocalyx Treatment Compositions and Methods.

[107] Hippensteel J.A., Uchimido R., Tyler P.D., Burke R.C., Han X., Zhang F., McMurtry S.A., Colbert J.F., Lindsell C.J., Angus D.C. (2019). Intravenous fluid resuscitation is associated with septic endothelial glycocalyx dendothelial glycocalyx radation. Crit. Care.

[108] Banerjee S., Mwangi J.G., Stanley T.K., Mitra R., Ebong E.E. (2021). Rendothelial glycocalyx eneration and Assessment of the Endothelial glycocalyx To Address Cardiovascular Disease. Ind. Eng. Chem. Res..

[109] Barelli S., Alberio L. (2018). The role of plasma transfusion in massive bleeding: Protecting the endothelial glycocalyx?. Front. Med..

[110] Aldecoa C., Llau J.V., Nuvials X., Artigas A. (2020). Role of albumin in the preservation of endothelial glycocalyx intendothelial glycocalyx rity and the microcirculation: A review. Ann. Intensive Care.

[111] Kaur G., Harris N.R. (2023). Endothelial glycocalyx in retina, hyperglycemia, and diabetic retinopathy. Am. J. Physiol. Cell Physiol..

[112] Cooper S., Teoh H., Campeau M.A., Verma S., Leask R.L. (2019). Empagliflozin restores the intendothelial glycocalyx rity of the endothelial glycocalyx in vitro. Mol. Cell Biochem..

[113] Chia P.Y., Teo A., Yeo T.W. (2020). Overview of the Assessment of Endothelial Function in Humans. Front. Med..

[114] Sardu C., Paolisso P., Sacra C., Mauro C., Minicucci F., Portoghese M., Rizzo M.R., Barbieri M., Sasso F.C., D’Onofrio N. (2019). Effects of Metformin Therapy on Coronary Endothelial Dysfunction in Patients with Prediabetes with Stable Angina and Nonobstructive Coronary Artery Stenosis: The CODYCE Multicenter Prospective Study. Diabetes Care.

[115] Nafisa A., Gray S.G., Cao Y., Wang T., Xu S., Wattoo F.H., Barras M., Cohen N., Kamato D., Little P.J. (2018). Endothelial function and dysfunction: Impact of metformin. Pharmacol. Ther..

[116] Targosz-Korecka M., Malek-Zietek K.E., Kloska D., Rajfur Z., Stepien E.Ł., Grochot-Przeczek A., Szymonski M. (2020). Metformin attenuates adhesion between cancer and endothelial cells in chronic hyperglycemia by recovery of the endothelial glycocalyx barrier. Biochim. Biophys. Acta Gen. Subj..

[117] He Z., Du X., Wu Y., Hua L., Wan L., Yan N. (2019). Simvastatin promotes endothelial dysfunction by activating the Wnt/betacatenin pathway under oxidative stress. Int. J. Mol. Med..

[118] Song J.W., Zullo J.A., Liveris D., Dragovich M., Zhang X.F., Goligorsky M.S. (2017). Therapeutic Restoration of Endothelial glycocalyx in Sepsis. J. Pharmacol. Exp. Ther..

[119] Uchimido R., Schmidt E.P., Shapiro N.I. (2019). The glycocalyx: A novel diagnostic and therapeutic target in sepsis. Crit. Care.

[120] Triantafyllou C., Nikolaou M., Ikonomidis I., Bamias G., Kouretas D., Andreadou I., Tsoumani M., Thymis J., Papaconstantinou I. (2021). Effects of Anti-Inflammatory Treatment and Surgical Intervention on Endothelial glycocalyx, Peripheral and Coronary Microcirculatory Function and Myocardial Deformation in Inflammatory Bowel Disease Patients: A Two-Arms Two-Stage Clinical Trial. Diagnostics.

[121] Fuchs A., Groß S., Neumann T., Illerhaus A., Vink H., Klasen G., Gathof B., Annecke T. (2021). Immediate effects of whole blood donation on the endothelial surface layer and glycocalyx shedding. Blood Transfus..

[122] Ikonomidis I., Pavlidis G., Katsimbri P., Lambadiari V., Parissis J., Andreadou I., Tsoumani M., Boumpas D., Kouretas D., Iliodromitis E. (2020). Tocilizumab improves oxidative stress and endothelial glycocalyx: A mechanism that may explain the effects of biological treatment on COVID-19. Food Chem. Toxicol..

[123] Ashry N.A., Abdelaziz R.R., Suddek G.M. (2020). The potential effect of imatinib against hypercholesterolemia induced atherosclerosis, endothelial dysfunction and hepatic injury in rabbits. Life Sci..

[124] Hayakawa M., Kudo D., Saito S., Uchino S., Yamakawa K., Iizuka Y., Sanui M., Takimoto K., Mayumi T., Ono K. (2016). Antithrombin supplementation and mortality in sepsis-induced disseminated intravascular coagulation: A multicenter retrospective observational study. Shock.

[125] Chappell D., Jacob M., Hofmann-Kiefer K., Bruegger D., Rehm M., Conzen P., Welsch U., Becker B.F. (2007). Hydrocortisone preserves the vascular barrier by protecting the endothelial glycocalyx. Anesthesiology.

[126] Chappell D., Jacob M., Hofmann-Kiefer K., Rehm M., Welsch U., Conzen P., Becker B.F. (2009). Antithrombin reduces shedding of the endothelial glycocalyx following ischaemia/reperfusion. Cardiovasc. Res..

[127] ElSaadani M., Ahmed S.M., Jacovides C., Lopez A., Johnson V.E., Kaplan L.J., Schwab C.W., Smith D.H., Pascual J.L. (2021). Antithrombin III ameliorates post-traumatic brain injury cerebral leukocyte mobilization enhancing recovery of blood brain barrier intendothelial glycocalyx rity. J. Trauma Acute Care Surg..

[128] Yini S., Heng Z., Xin A., Xiaochun M. (2015). Effect of unfractionated heparin on endothelial glycocalyx in a septic shock model. Acta Anaesthesiol. Scand..

[129] Qu J., Cheng Y., Wu W., Yuan L., Liu X. (2021). Glycocalyx Impairment in Vascular Disease: Focus on Inflammation. Front. Cell Dev. Biol..

[130] Oduah E.I., Linhardt R.J., Sharfstein S.T. (2016). Heparin: Past, Present, and Future. Pharmaceuticals.

[131] Karlsson K., Marklund S.L. (1987). Heparin-induced release of extracellular superoxide dismutase to human blood plasma. Biochem. J..

[132] Kim H.J., Kim E., Baek S.-H., Kim H.Y., Kim J.-Y., Park J., Choi E.-J. (2018). Sevoflurane did not show better protective effect on endothelial glycocalyx layer compared to propofol during lung resection surgery with one lung ventilation. J. Thorac. Dis..

[133] Maldonado F., Morales D., Gutierrez R., Barahona M., Cerda O., Caceres M. (2020). Effect of sevoflurane and propofol on tourniquet-induced endothelial damage: A pilot randomized controlled trial for knee-ligament surgery. BMC Anesthesiol..

[134] Kim N.Y., Kim K.J., Lee K.Y., Shin H.J., Cho J., Nam D.J., Kim S.Y. (2021). Effect of volatile and total intravenous anesthesia on syndecan-1 shedding after minimally invasive gastrectomy: A randomized trial. Sci. Rep..

[135] Fang F.Q., Sun J.H., Wu Q.L., Feng L.Y., Fan Y.X., Ye J.X., Gao W., He G.L., Wang W.J. (2021). Protective effect of sevoflurane on vascular endothelial glycocalyx in patients undergoing heart valve surgery: A randomised controlled trial. Eur. J. Anaesthesiol..

